# Bidirectional associations of problematic social media use and problematic gaming with mental health difficulties and strengths in adolescents: Sex and social support as potential moderators

**DOI:** 10.1111/jora.70076

**Published:** 2025-09-12

**Authors:** Luka Todorovic, Hanan Bozhar, Susanne R. de Rooij, Annabel Bogaerts, Bianca E. Boyer, Helle Larsen

**Affiliations:** ^1^ Department of Psychology University of Amsterdam Amsterdam The Netherlands; ^2^ Department of Public and Occupational Health, Amsterdam University Medical Center University of Amsterdam Amsterdam The Netherlands; ^3^ Centre for Urban Mental Health University of Amsterdam Amsterdam The Netherlands; ^4^ Department of Epidemiology and Data Science, Amsterdam University Medical Center University of Amsterdam Amsterdam The Netherlands; ^5^ Developmental and Educational Psychology Leiden University Leiden The Netherlands

**Keywords:** adolescence, cross‐lagged, mental health, problematic gaming, problematic social media use, sex, social support

## Abstract

The evidence on the direction of associations between mental health and problematic social media use (PSMU) and problematic gaming (PG) in adolescents remains inconclusive. Therefore, this study investigated a comprehensive model of temporal associations between mental health difficulties and strengths, and PSMU/PG, while accounting for sex and perceived social support as potential moderators. Mental health domains were measured with the Strengths and Difficulties Questionnaire, PSMU was measured with the Social Media Disorder Scale, and PG with the Internet Gaming Disorder Scale, assessed at two time points (2019, 2021). The analysis sample consisted of 645 Dutch adolescents (63% boys; *M*_age_ = 15.8, *SD* = 0.3, at Time 1) from the Amsterdam Born Children and their Development cohort. Cross‐lagged panel analysis did not indicate general bidirectional associations, but the multigroup analysis revealed separate temporal associations by sex and social support. In girls, emotional problems preceded PSMU and PG. In boys, PG preceded emotional problems and hyperactivity/inattention preceded PSMU. Adolescents with more perceived social support did not show a relationship between emotional problems and subsequent PG. We suggest that emotional problems may be a potential risk factor for PSMU/PG in girls, while they may be considered a negative consequence of PG in boys. Additionally, hyperactivity/inattention may be a risk factor for PSMU in boys, and social support may be a general protective factor for PG. These findings highlight the importance of understanding individual differences in the relationships between PSMU/PG and mental health symptoms.

## INTRODUCTION

New generations of adolescents are growing up engaged with social media and video games, which have been linked to mental health risks such as problematic social media use (PSMU) and problematic gaming (PG). PSMU refers to problematic use of social media apps such as Instagram, Snapchat, Facebook, and TikTok (Andreassen, 2015; Hendrikse & Limniou, 2024; Kircaburun et al., 2018), mostly via smartphone devices (Marino et al., 2021). PG refers to problematic use of online and offline video games, via devices such as computers and smartphones (Li et al., 2023; Paik et al., 2017). Both PSMU and PG are conceptualized as behavioral addictions. Prevalence of PSMU is around 5% in the general population, with adolescents showing higher prevalence rates than adults (Cheng et al., 2021): 9% among boys and 13% among girls (Boniel‐Nissim et al., 2024). Regarding PG, the prevalence is around 3% in the general population, with adolescents showing higher prevalence rates than adults (Kim et al., 2022): 16% among boys and 7% among girls (Boniel‐Nissim et al., 2024).

PSMU and PG are related constructs with notable similarities (Király et al., 2014; Sánchez‐Fernández et al., 2024). Symptoms of these problematic behaviors include salience (thinking and/or planning when to engage with the problematic activity again), tolerance (needing to engage more with the problematic activity), mood modification (engaging in the problematic activity in order to cope with emotional states), relapse (resorting to the problematic activity despite attempts to reduce it), withdrawal (experiencing unpleasant feelings when not engaging in the problematic activity), and conflict (experiencing negative impact of the problematic activity on different aspects of life; Gentile et al., 2017; Paulus et al., 2018). Unlike PSMU, PG is included as a mental health disorder in both the Diagnostic and Statistical Manual of Mental Disorders (5th ed.; DSM‐5; American Psychiatric Association, 2013) and International Classification of Diseases (11th ed.; ICD‐11; World Health Organization, 2019; Zhou et al., 2024). Studies show that PSMU and PG are correlated, with estimates of 0.25 in the general population (Andreassen et al., 2016; Chang et al., 2022; Henzel & Håkansson, 2021; Wong et al., 2020), up to ranges between 0.39 and 0.73 for adolescents and younger adults (Pontes, 2017; Reer et al., 2020). This seems to suggest that the affected users might be susceptible to simultaneous problems with multiple digital media. This makes simultaneous investigating of PSMU and PG relevant, as their distinct and shared associations with mental health could reveal whether they reflect common vulnerabilities or unique influences.

Digital media use has been associated with both mental health strengths and difficulties, illustrating that digital media can provide an environment for both flourishing and suffering during adolescence (Gudka et al., 2021; Law et al., 2019; Raith et al., 2021; West et al., 2024). Recently, there are ongoing debates in the scientific community about the interpretation of findings linking social media use to adolescents' mental health, where the point of contention is the directionality of associations between social media use and mental health (Odgers, 2024). In this paper, we use the term mental health as an umbrella term covering both adaptive and maladaptive intra‐ and interpersonal functioning across behavioral, emotional, and social domains of functioning. It is acknowledged that the claims about negative effects of social media use on adolescent mental health remain inconclusive due to a number of methodological shortcomings in this research area (Ghali et al., 2023; Odgers & Jensen, 2020; Orben, 2020; Valkenburg et al., 2022), including a lack of longitudinal studies (studies are mostly cross‐sectional), use of small and/or convenience samples (which reduces generalizability), and inconsistent measurement of digital media use (focus on duration of use and self‐reports). However, research on PSMU and PG has shown more robust associations with adverse mental health outcomes in adolescents (Boer et al., 2020; Hylkilä et al., 2024; Limone et al., 2023). To date, it still remains unclear whether digital media use is a cause, a consequence, or both in relation to mental health (Montag et al., 2024; Paulus et al., 2018; Shannon et al., 2022), as well as whether it is associated with positive and/or negative mental health outcomes, and under which circumstances.

From a developmental psychopathology perspective (Cicchetti & Rogosch, 2002) and in line with the I‐PACE model (Brand et al., 2016), PSMU and PG have been conceptualized as maladaptive coping strategies to regulate emotional and behavioral problems: adolescents with heightened emotional–behavioral problems may increasingly rely on digital media to escape distress, whereas greater adaptive regulation and social competencies may buffer against the development of such problems. Over time, excessive engagement in digital media use may, in turn, exacerbate mental health difficulties by reinforcing maladaptive regulation patterns. In addition, transactional models of development (Sameroff, 2009) highlight the importance of contextual factors in shaping developmental outcomes—for example, adolescents who increasingly rely on digital media for coping with difficulties may create unfavorable conditions in their social context, which could then further reinforce their problematic digital media use patterns.

### Domains of mental health and problematic digital media use

Research on emotional problems, such as symptoms of anxiety and depression, and problematic digital media use in adolescence has found positive associations with social media use, but methodological challenges leave the magnitude and practical significance of the relationship in question (Keles et al., 2019; Mundy et al., 2020; Valkenburg et al., 2022; Vidal et al., 2020). PSMU has been found to exhibit a moderate positive association with depressive symptoms, more so than time spent on social media and intensity of social media use (Cunningham et al., 2021). Similarly, PSMU has shown a moderate positive association with social anxiety, with a stronger relationship observed in men (Wu et al., 2024). Systematic reviews found a positive relationship between PSMU and both depression and anxiety in adolescents and adults, as well as between PG and social anxiety, suggesting the possibility of bidirectional effects (Gioia et al., 2022; Lopes et al., 2022). It was suggested that problematic digital media use may serve as a coping mechanism for emotion regulation difficulties (Gioia et al., 2021; Wartberg et al., 2021) or low perceived social support (Uçur & Dönmez, 2021) and further exacerbates these problems as a negative coping mechanism. A longitudinal study with university students found a bidirectional relationship between PSMU and anxiety, with PSMU also predicting later depression; meanwhile, PG was related to subsequent anxiety, and depression was related to subsequent PG (Chang et al., 2022); while that study had the advantage of using three waves over the duration of a year, it did not have an adolescent sample or investigate other mental health domains and individual differences.

Symptoms of Attention‐Deficit/Hyperactivity Disorder (ADHD) have also been related to problematic digital media use, with research frequently indicating a positive relationship. It was found that high frequencies of digital media use in adolescents are associated with subsequent symptoms of ADHD (Karen et al., 2018), although research remains inconclusive about the underlying mechanisms. Previous research has also indicated that adolescents with ADHD or symptoms thereof may tend to show more problematic digital media use (Kim et al., 2019; Werling et al., 2022), suggesting a bidirectional relationship between ADHD and problematic digital media use. Systematic reviews found a high prevalence of ADHD among adolescents with PG and emphasized the relationship between PG and symptoms of ADHD, especially symptoms of inattention (Coutelle et al., 2024; Männikkö et al., 2017). A literature review of longitudinal studies investigating the relationship between PSMU and ADHD in adolescents suggested a bidirectional relationship (Thorell et al., 2022). Bidirectional relationships between problematic internet use and hyperactivity/inattention were found in a Japanese population study of adolescents (Morita et al., 2021).

Conduct problems have been looked at in relation to problematic digital media use, with some research indicating positive associations. Aspects of social media use (socializing, shopping, connecting with strangers) have been linked to conduct problems in adolescents (De Calheiros Velozo & Stauder, 2018), and it was found that childhood conduct disorder and antisocial personality disorder are positively associated with social media use in emerging adults (Galica et al., 2017). Conduct problems have been found to exhibit a positive association with PG (Richard et al., 2020) and studies on associations of PG and PSMU with a variety of mental health problems reported that both are positively associated with conduct problems in adolescents (Mérelle et al., 2017), as well as with antisocial behavior (Wartberg & Kammerl, 2020). Moreover, intensive digital technology use has been associated with both same‐day and long‐term increases in conduct disorder symptoms, as well as poorer self‐regulation among adolescents at risk for mental health problems (George et al., 2017). While conduct problems highlight negative outcomes, examining prosocial behavior could provide a more balanced understanding of the potential impact of digital media use on adolescent mental health.

Prosocial behavior has been rarely looked at in the context of problematic digital media use. It is considered a mental health strength (Goodman, 1997) because it reflects social competence and empathy, and even though it can be considered a behavioral outcome, it is also recognized as a protective factor in adolescent development (Jung & Schröder‐Abé, 2019; Speyer et al., 2023). During adolescence, when peers and social connections become increasingly vital for mental health compared to childhood (Tomova et al., 2021), digital media have the potential to impact prosocial development in novel ways (Armstrong‐Carter & Telzer, 2021). There seems to be a positive relationship between social media use and (online) prosocial behavior in adolescents (Lysenstøen et al., 2021), while the same relationship in emerging adults seems complex and context‐dependent (Hui et al., 2024). There are findings from qualitative and quantitative research indicating that adolescents experience social media use as beneficial when it comes to exhibiting prosocial behavior (Bhadra & Kumar, 2023; Erreygers et al., 2016). A study with adolescents found a negative association between PG and prosocial behavior (García‐Gil et al., 2022), although no differences were observed in prosocial tendencies between adults with and without PG (Collins & Freeman, 2013). Overall, the relationship of prosocial behavior with problematic digital media use remains relatively unexplored, but there are indications that prosocial behavior is negatively associated with addiction problems regardless of the type of addiction (Esparza‐Reig et al., 2021).

### Sex and social support as potential moderators

The levels and trajectories of mental health symptoms significantly differ between boys and girls across adolescence (Murray et al., 2021). Boys are found to exhibit a higher prevalence of PG than girls (Fam, 2018; Mihara & Higuchi, 2017), likely due to a variety of relevant neurological and cultural factors which require further investigation (Marraudino et al., 2022). In addition, some studies found girls to exhibit a higher prevalence of PSMU than boys (Bouna‐Pyrrou et al., 2018; Su et al., 2020), although other recent studies found no sex differences in the prevalence of PSMU (Casale et al., 2023; Cheng et al., 2021; Meng et al., 2022). Beyond differences in prevalence, there is evidence that sex might moderate the relationships between mental health and digital media use. For example, girls were found to be more likely than boys to engage in social comparison and feedback‐seeking on social media platforms, which can intensify emotional problems (Nesi & Prinstein, 2015; Twenge & Martin, 2020). Boys were observed to more often show higher levels of impulsivity and sensation‐seeking in the context of gaming, which may increase their susceptibility to gaming problems, although the evidence is not clear on whether boys are at a higher risk of developing PG (Marraudino et al., 2022) or whether prolonged gaming relates to their emotional well‐being less than it does for girls (Lee et al., 2017). Overall, there is evidence to suggest that the relationship between mental health and PSMU and PG may differ between boys and girls.

In addition to individual factors such as sex, social context can also play an important role in how adolescent development relates to problematic digital media use. Perceived social support is a potentially important contextual factor, as it may influence how adolescents cope with emotional and behavioral challenges. From a developmental psychopathology perspective, and in line with the transactional model of development (Sameroff, 2009), such contextual factors shape whether individual predispositions result in maladaptive outcomes. For instance, perceived social support from family has been associated with lower levels of PSMU among adolescents, although this association did not extend to social support from friends (Lin et al., 2023). Among Chinese students, perceived social support has been found to act as a protective factor in the association between stress and problematic smartphone use, while online social support mediated this relationship (Zhao et al., 2021). The impact of online and offline social support may depend on its emotional quality, predicting either functional or problematic use among adolescents (Benvenuti et al., 2023) and students (Meshi & Ellithorpe, 2021). A recent meta‐analysis indicated that online social support was positively associated with PSMU, particularly among women (Ma et al., 2024), which suggests a potentially negative impact of social support. Low perceived social support was also linked to PG in adolescents (Meng et al., 2024; Uçur & Dönmez, 2021). Overall, these findings suggest that social support may be a relevant contextual factor in either attenuating or, in some cases, increasing PSMU and PG, depending on source (e.g., friends, family) and quality (e.g., online, offline). It may therefore moderate the relationships between adolescents' strengths and difficulties and their PSMU or PG.

### The present study

To address the aforementioned gaps in the literature, we investigated the longitudinal relationships of adolescents' PSMU and PG with mental health difficulties (emotional problems, hyperactivity/inattention, conduct problems) and strengths (prosocial behavior), and examined whether sex and social support were potential moderators of these relationships. All the variables were studied within a single model in order to account for shared variation between PSMU and PG and the mental health domains. This enabled us to compare the relative strength of associations of different mental health domains when it comes to both forms of problematic digital media use (and vice versa).

While previous research has provided partial indications about the significance and direction of these associations, there remains a lack of empirical longitudinal studies providing more robust evidence. The formulated hypotheses align with the expectations based on previous research and theory. Therefore, it was hypothesized that (I) mental health difficulties at T1 would be associated with more problematic digital media use at T2, (II) mental health strengths at T1 would be associated with less problematic digital media use at T2, (III) problematic digital media use at T1 would be associated with more mental health difficulties at T2, and (IV) problematic digital media use at T1 would be associated with less mental health strengths at T2. In addition, we expected that these associations may be moderated by sex and perceived social support.

## METHOD

### Participants

The community sample consisted of 2663 Dutch adolescents (54% girls). Their data were retrieved from the Amsterdam Born Children and their Development (ABCD) cohort study (Van Eijsden et al., 2010) and included responses from two time points: Time 1 (2019–2020; T1) and Time 2 (2021; T2). Most of the sample (81%) was enrolled in general or higher achievement secondary education. The inclusion criterion for the main analysis was that participants reported both PSMU and PG at both T1 and T2 (to avoid imputing values for participants who did not engage in any digital behavior at all). There were no predefined exclusion criteria for the current study. This resulted in an analysis sample of 645 participants. The analysis sample had an average age of 15.8 (*SD* = 0.3) at T1 and 17.4 (*SD* = 0.3) at T2, and it consisted of 411 boys (64%) and 234 girls (36%).

### Procedure

We used data from the fifth and sixth waves of the ABCD study (Van Eijsden et al., 2010). This longitudinal study started in 2003–2004 when all pregnant women living in Amsterdam were invited to participate in the cohort study. Data was collected through digital (smartphone app, website link) or paper questionnaires. Completing the questionnaires took approximately 50 min, with the possibility to fill out the different thematic parts across multiple takes. Rosenberg self‐esteem scale was omitted from the study due to measurement error. During both waves, the participants received a webstore voucher worth €10 upon completing the questionnaires. The Medical Ethics Review Committee of the [ANONYMOUS] concluded that a full review and official approval of this study was not required according to Dutch law (reference decisions [ANONYMOUS] and [ANONYMOUS]). All participants provided written informed consent, and for those aged 16 or younger, both parental consent and the child's assent were obtained.

### Measures

#### Social Media Disorder Scale

PSMU was measured with the Social Media Disorder Scale (Van Den Eijnden et al., 2016). It is a validated instrument which includes 9 items answered with binary (“yes/no”) response options. The items inquire about how the participant felt during the past year in relation to social media use (e.g., “During the past year, have you often felt bad when you could not use social media?”). The total score is calculated as a sum of scored responses and ranges between 0 and 9, with a proposed cutoff from ≥5 indicating problematic use. Internal consistency of the scale was good, with Cronbach's alpha between *α* = .76 and *α* = .82 across three Dutch adolescent samples (Van Den Eijnden et al., 2016), and *α* = .56 (T1)–.52 (T2) in the current study.

#### Internet Gaming Disorder Scale

PG was measured with the Internet Gaming Disorder Scale (Pontes & Griffiths, 2015). It is a validated instrument which includes 9 items answered on a 5‐point Likert scale, but versions with binary (“yes/no”) response options which we used in the study are also reliable and valid (Lemmens et al., 2015). The items inquire about how the participant felt during the past year in relation to gaming (e.g., “Do you systematically fail when trying to control or cease your gaming activity?”). The total score is calculated as a sum of scored responses and ranges between 0 and 9 and the proposed cutoff was >6. Internal consistency of the scale was good, with Cronbach's alpha between *α* = .87 and *α* = .88 across three adult samples (Pontes & Griffiths, 2015), and *α* = .72 (T1)–.57 (T2) in the current study.

#### Strengths and Difficulties Questionnaire

Mental health strengths and difficulties were measured with the Strengths and Difficulties Questionnaire (Goodman, 1997) which is a validated instrument including 25 items answered on a 3‐point Likert scale (“Not True; Somewhat True; Certainly True”). The total scores are calculated as a sum of scored responses. The emotional symptoms subscale contains 5 items, which inquire about how the participant felt in the past 6 months regarding symptoms of anxiety and depression (e.g., “I worry a lot”). Internal consistency of the subscale was somewhat satisfactory, with Cronbach's alpha *α* = .66 in a sample of youth (Goodman, 2001), and *α* = .73 (T1)–.76 (T2) in the current study. The hyperactivity/inattention subscale contains 5 items, which inquire about how the participant felt in the past 6 months regarding symptoms of ADHD (e.g., “I am restless, I cannot stay still for long.”). Internal consistency of the subscale was somewhat satisfactory, with Cronbach's alpha *α* = .67 in a sample of youth (Goodman, 2001), and *α* = .69 (T1)–.68 (T2) in the current study. The conduct problems subscale contains 5 items, which inquire about how the participant felt in the past 6 months regarding conduct problems (e.g., “I get very angry and often lose my temper”). Internal consistency of the subscale was somewhat unsatisfactory, with Cronbach's alpha *α* = .60 in a sample of youth (Goodman, 2001), and *α* = .40 (T1)–.43 (T2) in the current study. Finally, the prosocial behavior subscale contains 5 items, which inquire about how the participant felt in the past 6 months regarding behavior toward others (e.g., “I am helpful if someone is hurt, upset, or feeling ill”). Internal consistency of the subscale was somewhat satisfactory, with Cronbach's alpha *α* = .66 in a sample of youth (Goodman, 2001), and *α* = .57 (T1)–.54 (T2) in the current study.

#### Multidimensional Scale of Perceived Social Support

Social support was measured with the Multidimensional Scale of Perceived Social Support (MSPSS; Zimet et al., 1988). The MSPSS is a validated instrument, which includes 12 items answered on a 7‐point Likert scale (ranging from “Strongly Disagree” to “Strongly Agree”). The items inquire about how the participant feels about relationships with family, friends, and significant others (e.g., “I get the emotional help and support I need from my family”). The total score is calculated as a sum of scored responses. Internal consistency of the subscale was good, with Cronbach's alpha *α* = .86 in a sample of youth (Bruwer et al., 2008), and *α* = .80 (T1)–.84 (T2) in the current study.

### Statistical analysis plan

**FIGURE 1:**
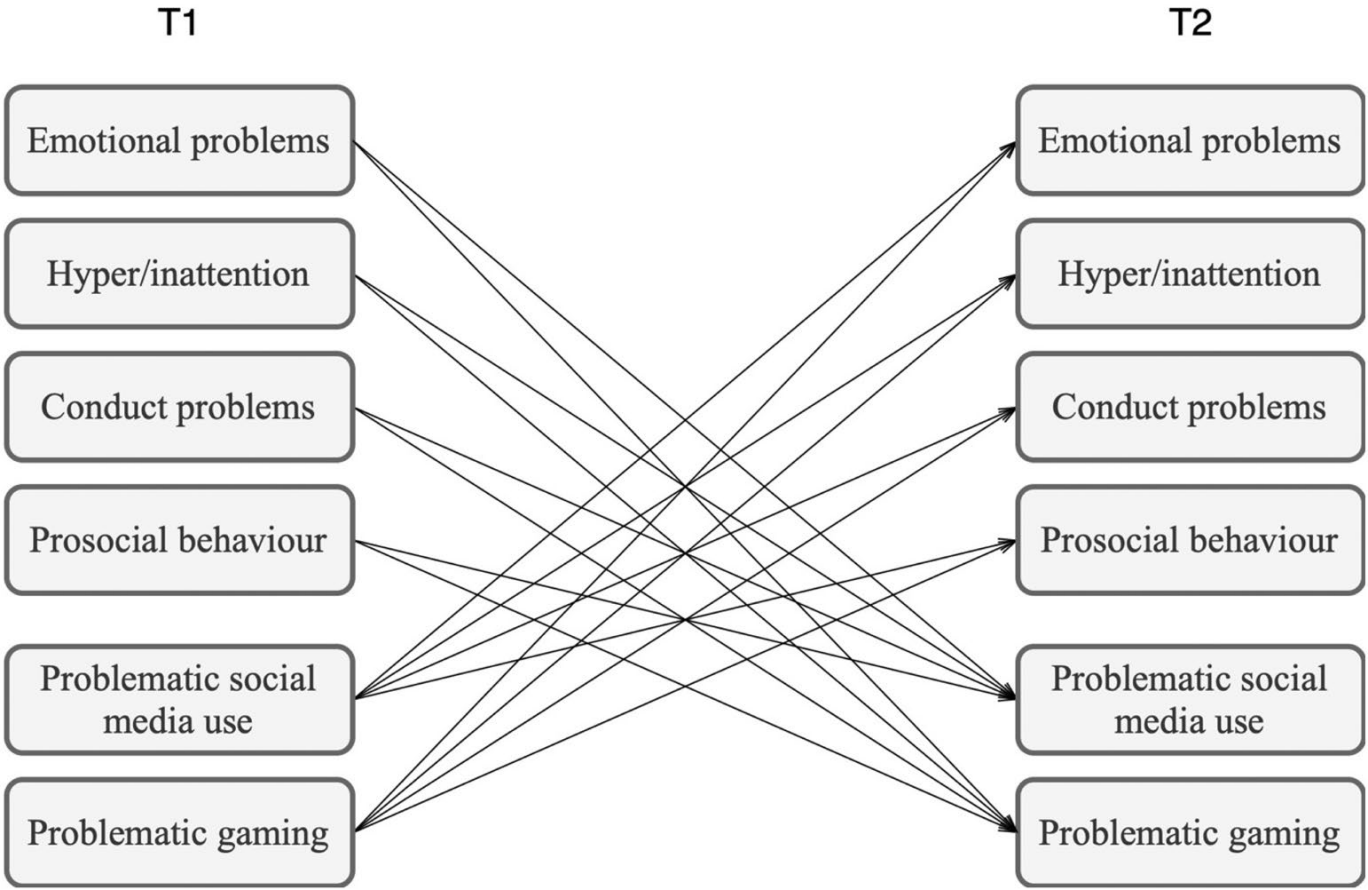
Cross‐lagged panel model (conceptual model). T1 = Time 1; T2 = Time 2. The paths relevant to hypothesis testing (i.e., cross‐lagged paths) are shown. Autoregressive paths from T1 to T2 and within‐time paths between variables at T1 and T2 are omitted.

Analyses were conducted in RStudio (Posit Team, 2024). Before performing the main analysis, we conducted an a priori power analysis, investigated the missing data, and tested the model assumptions (measurement invariance, multicollinearity, linearity, homoscedasticity, and normality). The main analysis was a cross‐lagged panel model (CLPM), which simultaneously estimates the associations of all the main variables in the model (see Figure 1) and allows for testing temporal precedence and directionality in longitudinal data (Baribeau et al., 2022). The analysis was conducted with continuous variables. In a first step, we applied CLPM with problematic digital media use (PSMU and PG) and mental health difficulties (emotional problems, hyperactivity/inattention, conduct problems) using the *lavaan* package (Rosseel, 2012). In a second step, we added mental health strengths (prosocial behavior) to examine whether the model significantly changed. Next, the main analysis was repeated with the entire sample after we used multiple imputation using the *mice* package (van Buuren & Groothuis‐Oudshoorn, 2011) to account for missing data. The imputation procedure was based on multivariate models that included all of the model variables, and the imputation method was random forest with 50 imputation cycles. Finally, we conducted multigroup CLPM to investigate the potential effects of sex and social support.

#### Power analysis

An a priori power analysis was conducted in RStudio (Posit Team, 2024) using the *semPower* package (Moshagen & Bader, 2023), and it indicated that 239 participants were necessary to observe medium (0.3) cross‐lagged effects with good power (0.8) and that 2227 participants were necessary to observe small (0.1) cross‐lagged effects with good power (0.8). An iterative a priori power analysis cycle indicated that 645 participants were enough to observe small‐to‐medium (0.19) cross‐lagged effects with good power (0.8).

#### Missing data

After the data from the two waves were merged it was observed that from the total of 2663 participants, 1458 participated in both waves. Together with participants who did not provide responses, this resulted in missing data on the study variables, at T1 and T2, respectively: 16% and 33% for emotional problems, hyperactivity/inattention, conduct problems, and prosocial behavior; 26% and 32% for social support; 32% and 38% for PSMU; and 32% and 62% for PG. The chance of observing missing data on PSMU and PG overlapped with answering negatively on the screener items inquiring whether participants use social media/video games at all (e.g., “Do you play (online) games?”). In case of a negative answer, the participants were not offered to complete the PSMU/PG questionnaire. This indicates that the data were missing for nonusers of either form of digital media, along with participants who dropped out for other reasons. Systematic missingness for nonusers of digital media suggests that some data is missing not at random (MNAR), requiring caution in interpreting multiple imputation results.

Comparisons between the full sample (*N* = 2663) and the analysis sample (*n* = 645) revealed significant differences on several measures. The gender distribution differed significantly between samples, *χ* ^2^(1) = 67.31, *p* < .001, with the analysis sample having a higher proportion of boys (increased from 46% to 64%) than the full sample. In addition, the analysis sample showed significantly higher levels of PG, *t*(1022) = −5.40, *p* < .001, and significantly lower levels of emotional problems, *t*(1175) = 2.31, *p* = .021, conduct problems, *t*(1230) = 2.28, *p* = .023, and prosocial behavior, *t*(1056) = 3.02, *p* = .003, compared to the full sample. These differences align with the observed sex composition differences, as boys—who are overrepresented in the analysis sample—typically report higher PG (Fam, 2018; Mihara & Higuchi, 2017) and differ in mental health symptoms compared to girls (Murray et al., 2021).

#### Model assumptions

To test measurement invariance of the model variables across time, we fit a configural model and examined its fit indices (CFI = 0.987; RMSEA = 0.053; TLI = 0.941; SRMR = 0.025), which indicated good fit compared to standard critical values (CFI ≥ 0.95, RMSEA ≤ 0.06, TLI ≥ 0.95, SRMR ≤ 0.08; Hu & Bentler, 1999), meaning that the basic factor structure was consistent across time. We then compared the configural model to a metric model. There was no significant difference between the models, which suggested that factor loadings remained invariant across time, indicating that metric invariance was observed. Comparing the metric and scalar models indicated differences, *χ* ^2^(6) = 433, *p* < .001, indicating that scalar invariance was not observed. Overall, the crucial measurement invariance assumptions were observed, although the lack of scalar invariance requires caution when interpreting mean differences across time. We also assessed multicollinearity by looking into the variance inflation factors of all the main model variables and found them to be within acceptable ranges (1.06–1.30), suggesting that multicollinearity was not an issue. Visual inspection of plots did not indicate deviations from linearity. When investigating homoscedasticity, we found that some predictor‐outcome pairs indicated that there was heteroscedasticity, so we used robust standard errors which ensured homoscedasticity of the residuals. Robust standard errors were used to account for the violation of normality of residuals, as identified by Shapiro–Wilk tests, with the relatively large sample size further ensuring the reliability of the results despite this issue.

## RESULTS

Table 1 presents the average levels of PSMU and PG in the sample, which were nonproblematic, and the average levels of mental health variables, which were nonproblematic. PSMU and PG were somewhat correlated at T1 (*r* = .15) and T2 (*r* = .24; see Table S6).

**TABLE 1:** Means (*M*) and standard deviations (*SD*) of model variables.

	*M* (*SD*)_T1_	*M* (*SD*)_T2_	Cutoff	Scale
PSMU	1.21 (1.40)	1.19 (1.28)	≥5	0–9
PG	0.94 (1.42)	0.72 (1.12)	≥5	0–9
Emotional problems	2.81 (2.35)	3.41 (2.57)	≥5	0–10
Hyperactivity/inattention	4.20 (2.47)	4.47 (2.46)	≥6	0–10
Conduct problems	1.46 (1.31)	1.47 (1.27)	≥4	0–10
Prosocial behavior	8.01 (1.50)	8.03 (1.59)	≤6	0–10
Social support	73.19 (8.97)	71.40 (9.51)	N/A	12–84

*Note*: T1 = Time 1; T2 = Time 2. Cutoff indicates elevated/problematic levels.

### Main analysis

For the main analysis, we first fit a CLPM that included mental health difficulties, and then compared it to the CLPM that also included prosocial behavior as a mental health strength. The comparison indicated no significant differences in model fit, *χ* ^2^(6) = 7.51, *p* = .27, so we proceeded using the more comprehensive model including both difficulties and strengths for the remainder of the analyses. This model had good model fit indices (CFI = 0.987; RMSEA = 0.053). The results indicated that all the autoregressive coefficients were significant, indicating that the score on each variable at T1 was related to its score on T2 (see Table 2). The cross‐lagged coefficient between emotional problems at T1 and PSMU at T2 was significant (β = .172, *p* < .001) as well as the cross‐lagged coefficient between emotional problems at T1 and PG at T2 (β = .152, *p* < .001). The cross‐lagged coefficients between other variables were not statistically significant. The results of the same analysis with the imputed data set generally supported these findings (see Table S1).

**TABLE 2:** Cross‐lagged panel model (*n* = 645).

	*B*	SE	*z*	*p*	β
Autoregressive paths					
Emotional problems T2					
~Emotional problems T1	0.717	0.030	23.674	<.001	.666
Conduct problems T2					
~Conduct problems T1	0.488	0.038	12.711	<.001	.501
Hyper/inattention T2					
~Hyper/inattention T1	0.668	0.027	24.429	<.001	.679
Prosocial behavior T2					
~Prosocial behavior T1	0.547	0.041	13.488	<.001	.517
PSMU T2					
~PSMU T1	0.280	0.042	6.681	<.001	.307
PG T2					
~PG T1	0.337	0.039	8.666	<.001	.426
Cross‐lagged paths					
Emotional problems T2					
~PSMU T1	0.012	0.058	0.207	.836	.007
~PG T1	−0.051	0.054	−0.948	.343	−.029
Conduct problems T2					
~PSMU T1	0.048	0.035	1.367	.172	.052
~PG T1	0.035	0.034	1.037	.300	.039
Hyper/inattention T2					
~PSMU T1	0.044	0.053	0.819	.413	.025
~PG T1	−0.050	0.051	−0.970	.332	−.029
Prosocial behavior T2					
~PSMU T1	−0.071	0.041	−1.718	.086	−.063
~PG T1	−0.061	0.041	−1.480	.139	−.055
PSMU T2					
~Emotional problems T1	0.093	0.022	4.183	<.001	.172
~Conduct problems T1	−0.002	0.043	−0.035	.972	−.002
~Hyper/inattention T1	0.002	0.021	0.080	.937	.003
~Prosocial behavior T1	−0.028	0.029	−0.985	.325	−.034
PG T2					
~Emotional problems T1	0.072	0.021	3.414	<.001	.152
~Conduct problems T1	0.037	0.037	0.983	.326	.043
~Hyper/inattention T1	0.000	0.022	0.021	.983	.001
~Prosocial behavior T1	−0.014	0.028	−0.485	.628	−.018

*Note*: T1 = Time 1; T2 = Time 2.

### Exploratory analysis

**FIGURE 2:**
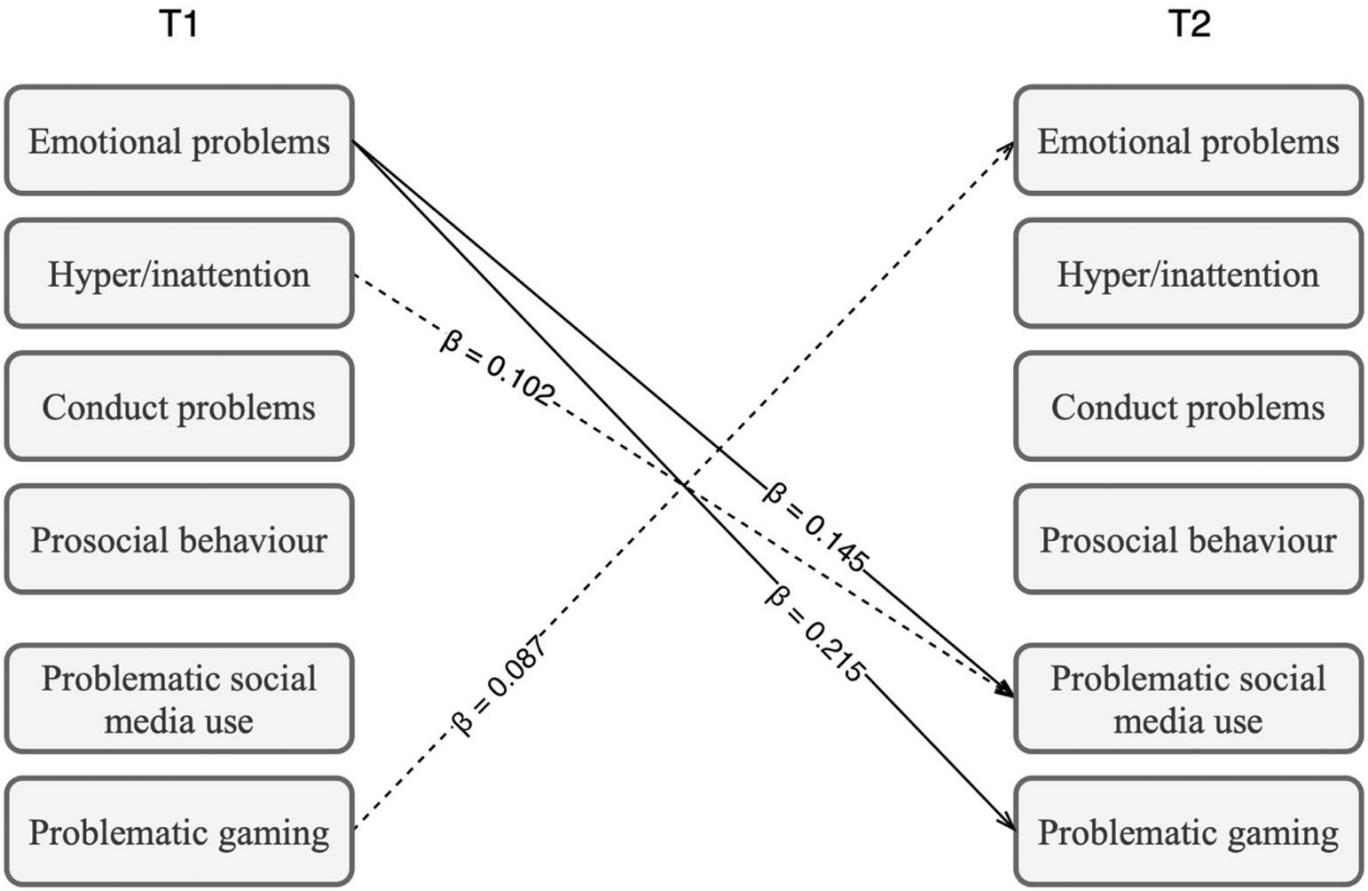
Significant paths of the multigroup cross‐lagged panel model by sex. T1 = Time 1; T2 = Time 2. Full arrows = female group. Dashed arrows = male group. Only the significant paths are shown. Nonsignificant cross‐lagged paths, autoregressive paths from T1 to T2, and within‐time paths between variables at T1 and T2 are omitted.

To explore sex differences, we conducted a multigroup analysis with the main CLPM by including sex as a group factor (see Figure 2). A chi‐squared test was conducted to compare the unconstrained and constrained multigroup CLPM, and the comparison indicated that the unconstrained model did not significantly improve model fit compared to the constrained model, *χ* ^2^(16) = 25.67, *p* = .058. However, given the large number of relationships in the model (including autoregressive and within‐time paths), an omnibus test could have been insensitive to practically meaningful sex differences, so we proceeded to further explore the potential sex differences in individual cross‐lagged paths. The multigroup CLPM had good model fit indices (CFI = 0.985; RMSEA = 0.050). Among girls, the cross‐lagged coefficient between emotional problems at T1 and PSMU at T2 was positive and significant (β = .145, *p* = .025), as well as the cross‐lagged coefficient between emotional problems at T1 and PG at T2 (β = .215, *p* = .001; see Supplement Table S2). Among boys, the cross‐lagged coefficient between PG at T1 and emotional problems at T2 was positive and significant (β = .087, *p* = .038), as was the cross‐lagged coefficient between hyperactivity/inattention at T1 and PSMU at T2 (β = .102, *p* = .048; see Supplement Table S3). The cross‐lagged coefficients between other variables were not statistically significant in either group.

To explore the moderating effect of perceived social support, we conducted a multigroup analysis with the main CLPM by including social support as a group factor. Participants were split into lower and higher support groups based on a tertile split of MSPSS scores at T1, where the lowest 33% were categorized as the lower group (*n* = 215; *M* = 63.2, *SD* = 7.69) and the remaining as the higher group (*n* = 430; *M* = 78.2, *SD* = 4.04). A chi‐squared test was conducted to compare the unconstrained and constrained multigroup CLPM, and the comparison indicated that the unconstrained model did not significantly improve model fit compared to the constrained model, *χ* ^2^(16) = 13.67, *p* = .623. However, as with sex differences, the omnibus test may have been insensitive to practically meaningful differences across groups, given the large number of relationships tested in the model. Therefore, we proceeded to examine the cross‐lagged coefficients separately by group (i.e., lower support vs. higher support). The multigroup CLPM showed good model fit indices (CFI = 0.990; RMSEA = 0.042). In the lower support group, emotional problems at T1 significantly predicted both PSMU (β = .140, *p* = .030) and PG (β = .223, *p* < .001) at T2 (see Table S4). In the higher support group, emotional problems at T1 significantly predicted PSMU at T2 (β = .183, *p* < .001), but the association with PG was not statistically significant (β = .076, *p* = .089; see Table S5). No other cross‐lagged paths reached significance in either group.

## DISCUSSION

This study investigated bidirectional associations of PSMU and PG with mental health difficulties and strengths among adolescents using a longitudinal design. The domains of mental health that were investigated were emotional problems, hyperactivity/inattention, conduct problems, and prosocial behavior, accounting for both mental health difficulties and strengths. Additionally, we explored the impact of sex and perceived social support on these associations. No overall bidirectional associations were observed, but different patterns for boys and girls emerged, and there were differences in the association between emotional problems and PG dependent upon the level of perceived social support.

The findings provide insight about temporal ordering between associations of problematic digital media use and mental health difficulties in adolescence, which is one of the key questions raised in previous research. The results of the main model showed that adolescents with more emotional problems at the start of the study were more likely to engage in both PSMU and PG, more than a year later. This finding supported the hypothesis that mental health difficulties would be associated with subsequent PSMU and PG. The effect sizes, as indicated by standardized coefficients, showed these to be small‐to‐medium effects. These findings align with previous research that found emotional problems to be an important factor in the context of PSMU (Lopes et al., 2022) and PG (Gioia et al., 2022). However, we did not find evidence for the relationships in the reverse direction, from PSMU and PG at T1 to emotional problems at T2.

These findings do not roughly align with some previous research which found PSMU and PG in university students to be associated with subsequent emotional problems (Chang et al., 2022). Overall, these results indicated the relevance of emotional problems as a potential risk factor for PSMU and PG, which might indicate a shared underlying mechanism of development for both types of problematic behavior. Such a pattern aligns with the I‐PACE model (Brand et al., 2016), which suggests that individuals with emotional vulnerabilities would be more likely to exhibit maladaptive internet‐related behaviors as a means of self‐regulation. Similarly, from a developmental psychopathology perspective (Cicchetti & Rogosch, 2002), emotional problems during adolescence may increase the likelihood of using digital media as a coping strategy, which could exacerbate PSMU or PG over time.

Opposite to what we expected, the results of the main model indicated that the other cross‐lagged paths in the model were not statistically significant: hyperactivity/inattention, conduct problems, and prosocial behavior were not significantly associated with either subsequent PSMU or PG, nor were PSMU and PG significantly associated with subsequent hyperactivity/inattention, conduct problems, and prosocial behavior. This contrasts with previous research that found ADHD symptoms to be an important factor in the context of PSMU (Kim et al., 2019; Werling et al., 2022) and PG (Coutelle et al., 2024), conduct problems to be an important factor in the context of PSMU and PG (Mérelle et al., 2017), and prosocial behavior to be potentially negatively affected by addiction problems (Esparza‐Reig et al., 2021). However, the multivariate approach used in the current study may indicate that the contributions of these domains are less robust when they are considered along with emotional problems. These findings also somewhat align with research which found that increased PSMU does not predict overall lower life satisfaction in adolescents (Li et al., 2024). Overall, these results suggest that emotional problems may be a key mental health difficulty related to PSMU and PG among adolescents, especially relative to other domains when modeled simultaneously.

Sex differences were explored with the multigroup model and indicated different patterns for girls and boys. For girls, two of the specified cross‐lagged paths in the model were significant: emotional problems at T1 were associated with PSMU and PG at T2. This finding mirrored the finding from the main model, and the effect sizes as indicated by standardized coefficients showed these to be small‐to‐medium effects. For boys, we found that PG at T1 was associated with emotional problems at T2, and hyperactivity/inattention at T1 was associated with PSMU at T2, and the effect sizes as indicated by standardized coefficients showed these to be small effects. These differences may reflect developmental processes, where girls tend to show more internalizing symptoms, and boys are more likely to externalize, perhaps even through behavior such as gaming. This may also relate to differences in maturational age (Graber, 2013), where earlier emotional and pubertal development among girls may explain why emotional problems precede problematic digital media use in girls, as they tend to encounter and navigate emotional challenges earlier than boys, potentially prompting earlier reliance on social media or gaming as coping mechanisms. For boys, hyperactivity/inattention might be another risk factor for PSMU, which now aligned with previous research that found ADHD symptoms to be an important factor in the context of PSMU (Kim et al., 2019; Werling et al., 2022), suggesting that PSMU may occur among boys who exhibit more impulsive or reward‐driven behavior. Overall, these results illustrate the relevance of considering sex differences when studying PSMU and PG, and their associations with mental health.

There was also a moderating effect of perceived social support. Adolescents with higher social support at T1 did not show a significant relationship between emotional problems at T1 and PG at T2, which might indicate that adolescents with higher perceived social support are less likely to use gaming as a negative coping mechanism for emotional problems and aligns with research linking low social support to increased PG risk (Meng et al., 2024; Uçur & Dönmez, 2021). However, no moderation was found for the relationship between emotional problems and PSMU at T2, contrasting with prior studies suggesting social support as a protective factor for PSMU (Meshi & Ellithorpe, 2021) and problematic smartphone use (Zhao et al., 2021). A possible explanation for this is that perceived social support measured both online and offline sources of support, as recent research suggests that online support seeking might reinforce rather than buffer PSMU (Ma et al., 2024). Overall, these findings support the idea that contextual factors such as social support may moderate whether mental health difficulties such as emotional problems associate with subsequent problems with digital media, such as PG.

### Limitations and strengths

Although we used a comprehensive model that allowed us to examine bidirectional associations between problematic digital media use and mental health, while accounting for individual differences based on sex and perceived social support, this study had several limitations, which could affect the generalizability and reliability of the findings. Regarding the study design, it would have been preferable to have more than two time points of assessment. This would have made it possible to use a random intercept cross‐lagged panel model, which provides more nuanced estimates by accounting for within‐person trait changes over time (Lucas, 2023). The current design does not provide results that can be used for causal inference, although it does provide a foundation for estimating the presence of bidirectional associations and the relative relevance of associations of the included variables. Regarding the study sample, it would have been preferable to have a sample of problematic users, meaning adolescents who experience clinically significant levels of PSMU and PG. Regarding measurement, PSMU, PG, and conduct problems subscales had notably lower internal consistency than in the validation samples (Goodman, 2001; Pontes & Griffiths, 2015; Van Den Eijnden et al., 2016), which could have increased measurement error and reduced power to detect effects. Regarding the measurement of the effects of social support, it also seems important for future studies in the field to differentiate between online and offline support and their different functions. In addition, the analysis sample only included participants who indicated both using social media and gaming, which might have limited generalizability (e.g., for adolescents who only use social media). Regarding the study context, it is noteworthy that the second assessment took place during the COVID‐19 pandemic, which may have influenced the findings in various ways, because it was a period of increased psychological distress for children and adolescents (Wolf & Schmitz, 2023).

This study also had several notable strengths that contribute to advancing our understanding of adolescent development and problematic digital media use. First, the longitudinal design moves beyond the predominantly cross‐sectional studies in the field, allowing for the examination of temporal associations and providing a foundation for future research into potential causal relationships. Second, the study simultaneously modeled multiple mental health domains (emotional problems, hyperactivity/inattention, conduct problems, and prosocial behavior) alongside two modalities of problematic digital media use, offering a nuanced perspective that accounts for shared variation between these constructs. Third, the inclusion of a broad community sample spanning middle to late adolescence ensures the findings are relevant for general populations of adolescents, enhancing the study's generalizability. Fourth, the use of multiple imputation for missing data strengthens the reliability of results by addressing potential biases due to incomplete data. Finally, the study explored contextual and individual differences, including sex‐specific patterns and the role of perceived social support, aligning with developmental frameworks that consider the complex interplay of individual and environmental factors during adolescence (Sameroff, 2009). Together, these methodological and conceptual strengths provide valuable insights into the risks and protective factors associated with PSMU and PG, with implications for improving adolescent well‐being.

## CONCLUSION

This study found no overall bidirectional associations between domains of mental health (i.e., emotional problems, hyperactivity/inattention, conduct problems, prosocial behavior) and problematic digital media use (i.e., social media and gaming) among adolescents. However, it was found that emotional problems preceded both PSMU and PG, indicating that emotional problems could be a major risk factor related to PSMU and PG in adolescence. Subsequent analyses uncovered that these relationships existed primarily among girls, while among boys, PG preceded emotional problems, showing an opposite pattern of temporal association compared to girls. In addition, hyperactivity/inattention preceded PSMU among boys, indicating that factors related to ADHD symptoms could be a risk factor for boys to develop PSMU. When it comes to social support, it was observed that the relationship between emotional problems and subsequent PG was only significant among adolescents with lower social support, indicating that PG may develop as a coping mechanism among adolescents with less social support. Future research should investigate these relationships and sex‐specific patterns further, with more extensive longitudinal designs, as well as in clinical samples of adolescent problematic digital media users, while accounting for both intra‐ and interindividual differences, in order to inform the development of targeted interventions that account for the underlying mental health vulnerabilities and promote healthier digital media engagement during adolescence.

## AUTHOR CONTRIBUTIONS

All listed authors have contributed to the manuscript substantially and have agreed to the final submitted version. Luka Todorovic: Conceptualization (lead), Methodology (lead), Data curation (equal), Validation (equal), Formal analysis (lead), Visualization (lead), Project administration (lead), Writing – original draft (lead), Writing – review and editing (equal). Hanan Bozhar: Data curation (equal), Investigation (equal), Validation (equal), Writing – review and editing (equal). Annabel Bogaerts: Conceptualization (equal), Writing – review and editing (equal). Bianca E. Boyer: Conceptualization (equal); Writing – review and editing (equal). Susanne R. de Rooij: Investigation (equal); Funding acquisition (equal); Writing – review and editing (equal). Helle Larsen: Conceptualization (equal), Investigation (equal), Supervision (lead), Funding acquisition (equal), Writing – review and editing (equal).

## FUNDING INFORMATION

The sixth wave of the data collection was funded by Centre for Urban Mental Health. They did not have an influence on the paper.

## CONFLICT OF INTEREST STATEMENT

The authors declare no conflicts of interest to disclose.

## ETHICS STATEMENT

Regarding the study that the data were obtained from The Medical Ethics Review Committee of the Academic Medical Centre Amsterdam concluded that a full review and official approval of that study was not required according to Dutch law (reference decisions W19_130#19.163 and W20_396#20.444).

## PATIENT CONSENT STATEMENT

All participants provided informed consent, and parent/guardian consent was obtained for participants for whom this was required by Dutch law.

## REGISTRATION

The study was registered with AsPredicted.org (identifier 156163).

## Supporting information


Table S1.
Table S2.
Table S3.
Table S4.
Table S5.
Table S6.

## Data Availability

Data sharing is not applicable to this article as no new data were created or analyzed in this study. Analytic script and model outputs of the study are publicly available at https://github.com/lukatodorovic30/ABCD‐study‐analysis.git.
