# Supplementary material for: Bidirectional associations of problematic social media use and problematic gaming with mental health difficulties and strengths in adolescents: Sex and social support as potential moderators
Source: J Res Adolesc. 2025 Sep 12;35(3):e70076. doi: 10.1111/jora.70076 (PMC12432349; doi:10.1111/jora.70076)
Supplement: Supplementary file 1 — Table S1. Table S2. Table S3. Table S4. Table S5. Table S6. [file JORA-35-0-s001.docx]

**Supplement**

**Table S1**

*Cross-lagged Panel Model with Imputed Data (n = 2663)*

|  | *B* | *SE* | 95% CI | *z* | *p* | β |
| --- | --- | --- | --- | --- | --- | --- |
| Autoregressive paths |  |  |  |  |  |  |
| emotional problems T2 |  |  |  |  |  |  |
| ~ emotional problems T1 | 0.650 | 0.025 | [0.602, 0.698] | 26.39 | <.001 | .593 |
| conduct problems T2 |  |  |  |  |  |  |
| ~ conduct problems T1 | 0.407 | 0.022 | [0.365, 0.450] | 18.69 | <.001 | .409 |
| hyper/inattention T2 |  |  |  |  |  |  |
| ~ hyper/inattention T1 | 0.581 | 0.023 | [0.535, 0.626] | 24.93 | <.001 | .585 |
| prosocial behaviour T2 |  |  |  |  |  |  |
| ~ prosocial behaviour T1 | 0.473 | 0.026 | [0.422, 0.523] | 18.38 | <.001 | .468 |
| PSMU T2 |  |  |  |  |  |  |
| ~ PSMU T1 | 0.269 | 0.023 | [0.225, 0.314] | 11.78 | <.001 | .294 |
| PG T2 |  |  |  |  |  |  |
| ~ PG T1 | 0.262 | 0.025 | [0.212, 0.311] | 10.38 | <.001 | .306 |
| Cross-lagged paths |  |  |  |  |  |  |
| emotional problems T2 |  |  |  |  |  |  |
| ~ PSMU T1 | 0.145 | 0.037 | [0.073, 0.217] | 3.97 | <.001 | .080 |
| ~ PG T1 | -0.097 | 0.040 | [-0.175, -0.019] | -2.44 | .015 | -.046 |
| conduct problems T2 |  |  |  |  |  |  |
| ~ PSMU T1 | 0.072 | 0.022 | [0.030, 0.115] | 3.34 | .126 | .080 |
| ~ PG T1 | 0.039 | 0.025 | [-0.011, 0.089] | 1.53 | .126 | .037 |
| hyper/inattention T2 |  |  |  |  |  |  |
| ~ PSMU T1 | 0.087 | 0.036 | [0.017, 0.158] | 2.43 | .015 | .051 |
| ~ PG T1 | -0.014 | 0.038 | [-0.089, 0.061] | -0.37 | .714 | -.007 |
| prosocial behaviour T2 |  |  |  |  |  |  |
| ~ PSMU T1 | -0.042 | 0.022 | [-0.085, 0.002] | -1.89 | .059 | -.039 |
| ~ PG T1 | -0.106 | 0.029 | [-0.163, -0.049] | -3.66 | <.001 | -.085 |
| PSMU T2 |  |  |  |  |  |  |
| ~ emotional problems T1 | 0.093 | 0.013 | [0.067, 0.118] | 7.20 | <.001 | .168 |
| ~ conduct problems T1 | 0.008 | 0.026 | [-0.042, 0.059] | 0.32 | .750 | .008 |
| ~ hyper/inattention T1 | 0.006 | 0.013 | [-0.020, 0.032] | 0.45 | .652 | .011 |
| ~ prosocial behaviour T1 | -0.011 | 0.023 | [-0.056, 0.034] | -0.48 | .634 | -.013 |
| PG T2 |  |  |  |  |  |  |
| ~ emotional problems T1 | 0.054 | 0.012 | [0.031, 0.077] | 4.56 | <.001 | .121 |
| ~ conduct problems T1 | 0.046 | 0.023 | [0.001, 0.091] | 1.96 | .050 | .057 |
| ~ hyper/inattention T1 | 0.006 | 0.012 | [-0.017, 0.030] | 0.54 | .590 | .015 |
| ~ prosocial behaviour T1 | -0.024 | 0.019 | [-0.062, 0.014] | -1.22 | .222 | -.034 |

*Note.* T1 = Time 1. T2 = Time 2.

**Table S2**

*Multigroup Cross-lagged Panel Model (Girls, n = 234)*

| Autoregressive paths | *B* | *SE* | *z* | *p* | β |
| --- | --- | --- | --- | --- | --- |
| emotional problems T2 |  |  |  |  |  |
| ~ emotional problems T1 | 0.607 | 0.034 | 17.999 | <.001 | .615 |
| conduct problems T2 |  |  |  |  |  |
| ~ conduct problems T1 | 0.488 | 0.033 | 14.977 | <.001 | .518 |
| hyper/inattention T2 |  |  |  |  |  |
| ~ hyper/inattention T1 | 0.664 | 0.028 | 23.564 | <.001 | .679 |
| prosocial behaviour T2 |  |  |  |  |  |
| ~ prosocial behaviour T1 | 0.534 | 0.036 | 14.732 | <.001 | .506 |
| PSMU T2 |  |  |  |  |  |
| ~ PSMU T1 | 0.225 | 0.034 | 6.667 | <.001 | .241 |
| PG T2 |  |  |  |  |  |
| ~ PG T1 | 0.337 | 0.030 | 11.226 | <.001 | .268 |
| Cross-lagged paths |  |  |  |  |  |
|  |  |  |  |  |  |
| emotional problems T2 |  |  |  |  |  |
| ~ PSMU T1 | -0.096 | 0.085 | -1.120 | .263 | -.060 |
| ~ PG T1 | 0.103 | 0.161 | 0.639 | .523 | .033 |
| conduct problems T2 |  |  |  |  |  |
| ~ PSMU T1 | 0.081 | 0.046 | 1.783 | .075 | .097 |
| ~ PG T1 | 0.157 | 0.086 | 1.824 | .068 | .097 |
| hyper/inattention T2 |  |  |  |  |  |
| ~ PSMU T1 | 0.139 | 0.078 | 1.772 | .076 | .085 |
| ~ PG T1 | -0.191 | 0.149 | -1.284 | .199 | -.061 |
| prosocial behaviour T2 |  |  |  |  |  |
| ~ PSMU T1 | -0.084 | 0.056 | -1.520 | .128 | -.083 |
| ~ PG T1 | -0.206 | 0.108 | -1.903 | .057 | -.105 |
| PSMU T2 |  |  |  |  |  |
| ~ emotional problems T1 | 0.083 | 0.037 | 2.241 | .025 | .145 |
| ~ conduct problems T1 | 0.110 | 0.073 | 1.512 | .131 | .105 |
| ~ hyper/inattention T1 | -0.044 | 0.037 | -1.174 | .241 | -.079 |
| ~ prosocial behaviour T1 | -0.057 | 0.059 | -0.974 | .330 | -.059 |
| PG T2 |  |  |  |  |  |
| ~ emotional problems T1 | 0.086 | 0.026 | 3.378 | <.001 | .215 |
| ~ conduct problems T1 | 0.044 | 0.050 | 0.872 | .383 | .060 |
| ~ hyper/inattention T1 | -0.002 | 0.026 | -0.087 | .930 | -.006 |
| ~ prosocial behaviour T1 | -0.061 | 0.041 | -1.468 | .142 | -.089 |

*Note.* T1 = Time 1. T2 = Time 2.

**Table S3**

*Multigroup Cross-lagged Panel Model (Boys, n = 411)*

| Autoregressive paths | *B* | *SE* | *z* | *p* | β |
| --- | --- | --- | --- | --- | --- |
| emotional problems T2 |  |  |  |  |  |
| ~ emotional problems T1 | 0.607 | 0.034 | 17.999 | <.001 | .559 |
| conduct problems T2 |  |  |  |  |  |
| ~ conduct problems T1 | 0.488 | 0.033 | 14.977 | <.001 | .492 |
| hyper/inattention T2 |  |  |  |  |  |
| ~ hyper/inattention T1 | 0.664 | 0.028 | 23.564 | <.001 | .678 |
| prosocial behaviour T2 |  |  |  |  |  |
| ~ prosocial behaviour T1 | 0.534 | 0.036 | 14.732 | <.001 | .498 |
| PSMU T2 |  |  |  |  |  |
| ~ PSMU T1 | 0.225 | 0.034 | 6.667 | <.001 | .266 |
| PG T2 |  |  |  |  |  |
| ~ PG T1 | 0.337 | 0.030 | 11.226 | <.001 | .452 |
| Cross-lagged paths |  |  |  |  |  |
|  |  |  |  |  |  |
| emotional problems T2 |  |  |  |  |  |
| ~ PSMU T1 | -0.079 | 0.069 | -1.145 | .252 | -.048 |
| ~ PG T1 | 0.113 | 0.054 | 2.074 | .038 | .087 |
| conduct problems T2 |  |  |  |  |  |
| ~ PSMU T1 | -0.036 | 0.046 | -0.774 | .439 | -.035 |
| ~ PG T1 | 0.050 | 0.036 | 1.378 | .168 | .062 |
| hyper/inattention T2 |  |  |  |  |  |
| ~ PSMU T1 | -0.097 | 0.073 | -1.337 | .181 | -.051 |
| ~ PG T1 | 0.020 | 0.057 | 0.357 | .721 | .014 |
| prosocial behaviour T2 |  |  |  |  |  |
| ~ PSMU T1 | -0.077 | 0.057 | -1.347 | .178 | -.060 |
| ~ PG T1 | -0.019 | 0.044 | -0.420 | .675 | -.019 |
| PSMU T2 |  |  |  |  |  |
| ~ emotional problems T1 | 0.006 | 0.028 | 0.202 | .840 | .010 |
| ~ conduct problems T1 | -0.053 | 0.040 | -1.311 | .190 | -.065 |
| ~ hyper/inattention T1 | 0.044 | 0.022 | 1.979 | .048 | .102 |
| ~ prosocial behaviour T1 | -0.053 | 0.034 | -1.561 | .119 | -.074 |
| PG T2 |  |  |  |  |  |
| ~ emotional problems T1 | 0.056 | 0.029 | 1.942 | .052 | .089 |
| ~ conduct problems T1 | 0.018 | 0.042 | 0.439 | .661 | .020 |
| ~ hyper/inattention T1 | 0.003 | 0.023 | 0.023 | .882 | .007 |
| ~ prosocial behaviour T1 | 0.018 | 0.035 | 0.035 | .608 | .022 |

*Note.* T1 = Time 1. T2 = Time 2.

**Table S4**

*Multigroup Cross-lagged Panel Model (Lower Social Support, n = 215)*

| Autoregressive paths | *B* | *SE* | *z* | *p* | β |
| --- | --- | --- | --- | --- | --- |
| emotional problems T2 |  |  |  |  |  |
| ~ emotional problems T1 | 0.733 | 0.032 | 22.720 | <.001 | .750 |
| conduct problems T2 |  |  |  |  |  |
| ~ conduct problems T1 | 0.490 | 0.034 | 14.626 | <.001 | .519 |
| hyper/inattention T2 |  |  |  |  |  |
| ~ hyper/inattention T1 | 0.672 | 0.028 | 23.685 | <.001 | .659 |
| prosocial behaviour T2 |  |  |  |  |  |
| ~ prosocial behaviour T1 | 0.523 | 0.036 | 14.471 | <.001 | .466 |
| PSMU T2 |  |  |  |  |  |
| ~ PSMU T1 | 0.268 | 0.035 | 7.727 | <.001 | .279 |
| PG T2 |  |  |  |  |  |
| ~ PG T1 | 0.334 | 0.027 | 12.519 | <.001 | .371 |
| Cross-lagged paths |  |  |  |  |  |
|  |  |  |  |  |  |
| emotional problems T2 |  |  |  |  |  |
| ~ PSMU T1 | -0.043 | 0.086 | -0.494 | .622 | -.023 |
| ~ PG T1 | -0.068 | 0.080 | -0.847 | .397 | -.038 |
| conduct problems T2 |  |  |  |  |  |
| ~ PSMU T1 | 0.044 | 0.060 | 0.734 | .463 | .043 |
| ~ PG T1 | -0.008 | 0.057 | -0.142 | .887 | -.008 |
| hyper/inattention T2 |  |  |  |  |  |
| ~ PSMU T1 | 0.069 | 0.091 | 0.761 | .446 | .038 |
| ~ PG T1 | -0.151 | 0.085 | -1.764 | .078 | -.087 |
| prosocial behaviour T2 |  |  |  |  |  |
| ~ PSMU T1 | -0.090 | 0.072 | -1.255 | .209 | -.074 |
| ~ PG T1 | -0.076 | 0.069 | -1.093 | .274 | -.065 |
| PSMU T2 |  |  |  |  |  |
| ~ emotional problems T1 | 0.070 | 0.032 | 2.165 | .030 | .140 |
| ~ conduct problems T1 | 0.044 | 0.057 | 0.772 | .440 | .050 |
| ~ hyper/inattention T1 | -0.004 | 0.035 | -0.127 | .899 | -.008 |
| ~ prosocial behaviour T1 | 0.012 | 0.053 | 0.217 | .828 | .013 |
| PG T2 |  |  |  |  |  |
| ~ emotional problems T1 | 0.110 | 0.031 | 3.541 | .001 | .223 |
| ~ conduct problems T1 | 0.071 | 0.055 | 1.288 | .198 | .083 |
| ~ hyper/inattention T1 | -0.007 | 0.034 | -0.199 | .842 | -.013 |
| ~ prosocial behaviour T1 | -0.022 | 0.052 | -0.420 | .674 | -.025 |

*Note.* T1 = Time 1. T2 = Time 2.

**Table S5**

*Multigroup Cross-lagged Panel Model (Higher Social Support, n = 430)*

| Autoregressive paths | *B* | *SE* | *z* | *p* | β |
| --- | --- | --- | --- | --- | --- |
| emotional problems T2 |  |  |  |  |  |
| ~ emotional problems T1 | 0.733 | 0.032 | 22.720 | <.001 | .624 |
| conduct problems T2 |  |  |  |  |  |
| ~ conduct problems T1 | 0.490 | 0.034 | 14.626 | <.001 | .479 |
| hyper/inattention T2 |  |  |  |  |  |
| ~ hyper/inattention T1 | 0.672 | 0.028 | 23.685 | <.001 | .689 |
| prosocial behaviour T2 |  |  |  |  |  |
| ~ prosocial behaviour T1 | 0.523 | 0.036 | 14.471 | <.001 | .508 |
| PSMU T2 |  |  |  |  |  |
| ~ PSMU T1 | 0.268 | 0.035 | 7.727 | <.001 | .305 |
| PG T2 |  |  |  |  |  |
| ~ PG T1 | 0.334 | 0.027 | 12.519 | <.001 | .464 |
| Cross-lagged paths |  |  |  |  |  |
|  |  |  |  |  |  |
| emotional problems T2 |  |  |  |  |  |
| ~ PSMU T1 | 0.037 | 0.069 | 0.539 | .590 | .021 |
| ~ PG T1 | -0.031 | 0.066 | -0.476 | .634 | -.017 |
| conduct problems T2 |  |  |  |  |  |
| ~ PSMU T1 | 0.045 | 0.036 | 1.248 | .212 | .053 |
| ~ PG T1 | 0.055 | 0.036 | 1.540 | .124 | .065 |
| hyper/inattention T2 |  |  |  |  |  |
| ~ PSMU T1 | 0.013 | 0.062 | 0.213 | .832 | .008 |
| ~ PG T1 | 0.007 | 0.060 | 0.125 | .901 | .004 |
| prosocial behaviour T2 |  |  |  |  |  |
| ~ PSMU T1 | -0.051 | 0.044 | -1.166 | .244 | -.049 |
| ~ PG T1 | -0.051 | 0.044 | -1.149 | .250 | -.048 |
| PSMU T2 |  |  |  |  |  |
| ~ emotional problems T1 | 0.106 | 0.027 | 3.878 | <.001 | .183 |
| ~ conduct problems T1 | -0.051 | 0.050 | -1.018 | .309 | -.048 |
| ~ hyper/inattention T1 | 0.008 | 0.024 | 0.327 | .744 | .016 |
| ~ prosocial behaviour T1 | -0.044 | 0.038 | -1.162 | .245 | -.051 |
| PG T2 |  |  |  |  |  |
| ~ emotional problems T1 | 0.036 | 0.021 | 1.703 | .089 | .076 |
| ~ conduct problems T1 | -0.016 | 0.040 | -0.408 | .683 | -.019 |
| ~ hyper/inattention T1 | 0.013 | 0.020 | 0.658 | .511 | .031 |
| ~ prosocial behaviour T1 | -0.001 | 0.030 | -0.016 | .987 | -.001 |

*Note.* T1 = Time 1. T2 = Time 2.

**Table S6**

*Correlation Table* *(n = 645)*

|  | 1. | 2. | 3. | 4. | 5. | 6. | 7. | 8. | 9. | 10. | 11. | 12. | 13. | 14. |
| --- | --- | --- | --- | --- | --- | --- | --- | --- | --- | --- | --- | --- | --- | --- |
| 1. T1 emotional problems | **1** | - | - | - | - | - | - | - | - | - | - | - | - | - |
| 2. T1 conduct problems | **.240** | **1** | - | - | - | - | - | - | - | - | - | - | - | - |
| 3. T1 hyper/inattention | **.331** | **.379** | **1** | - | - | - | - | - | - | - | - | - | - | - |
| 4. T1 prosocial behaviour | -.022 | **-.227** | **-.185** | **1** | - | - | - | - | - | - | - | - | - | - |
| 5. T1 social support | **-.276** | **-.276** | **-.195** | **.324** | **1** | - | - | - | - | - | - | - | - | - |
| 6. T1 PSMU | **.349** | **.184** | **.255** | **-.114** | **-.146** | **1** | - | - | - | - | - | - | - | - |
| 7. T1 PG | .010 | **.115** | **.118** | **-.187** | **-.128** | **.151** | **1** | - | - | - | - | - | - | - |
| 8. T2 emotional problems | **.678** | **.081** | **.231** | .032 | **-.122** | **.232** | -.021 | **1** | - | - | - | - | - | - |
| 9. T2 conduct problems | **.189** | **.513** | **.335** | **-.147** | **-.163** | **.151** | **.104** | **.174** | **1** | - | - | - | - | - |
| 10. T2 hyper/inattention | **.289** | **.271** | **.691** | -.071 | **-.101** | **.191** | .054 | **.327** | **.335** | **1** | - | - | - | - |
| 11. T2 prosocial behaviour | -.064 | **-.187** | **-.151** | **.534** | **.269** | **-.130** | **-.161** | -.036 | **-.182** | **-.090** | **1** | - | - | - |
| 12. T2 social support | **-.228** | **-.205** | **-.185** | **.256** | **.478** | **-.162** | -.055 | **-.274** | **-.239** | **-.173** | **.314** | **1** | - | - |
| 13. T2 PSMU | **.298** | **.089** | **.161** | -.056 | **-.128** | **.371** | .052 | **.365** | **.174** | **.239** | **-.100** | **-.197** | **1** | - |
| 14. T2 PG | **.178** | **.127** | **.129** | **-.101** | **-.190** | **.130** | **.435** | **.206** | **.128** | **.147** | **-.171** | **-.165** | **.239** | **1** |
| 15. Sex | **.434** | -.038 | **.083** | **.190** | .002 | **.258** | **-.316** | **.500** | .044 | **.126** | **.128** | -.053 | **.333** | -.056 |

*Note.* Point-biserial correlation was used for sex (0 = Boys, 1 = Girls). Significant correlations (*p* < **.05**) in bold.
